# Multi‐country foodborne event caused by cereulide in infant formula products 19 February 2026

**DOI:** 10.2903/j.efsa.2026.9984

**Published:** 2026-03-02

## Abstract

From 19 December 2025 to 13 February 2026, six EU countries (Austria, Belgium, Denmark, France, Luxembourg and Spain) and the United Kingdom reported infants with gastrointestinal symptoms and consumption of infant formula. Most presented with mild symptoms, but some hospitalisations occurred due to dehydration. The latest instance of disease onset was 6 February. Diagnostic challenges and limited surveillance are affecting Member States' ability to identify cases associated with this event.

In December 2025, food companies in multiple countries initiated the recall of several infant formula products across various brands and batches containing arachidonic acid oil ingredient contaminated with cereulide. On 2 February, EFSA published an assessment which estimated concerning levels of cereulide in infant formula. The recall within the EU was expanded and harmonised under a science‐based risk management approach, significantly reducing the likelihood of children's exposure to contaminated products in the EU.

Investigations are ongoing to identify cases which may be part of this event and verify if recalled batches, or other batches of infant formula products, served as the vehicle of illness. Belgium, Austria, Luxembourg and the UK reported infants with symptoms and the detection of cereulide in products consumed. Denmark, France and Spain reported observing symptomatic infants who had consumed products from recalled infant formula batches.

Symptoms of cereulide intoxication are generally mild, but infants under six months are more vulnerable to dehydration and electrolyte disturbances than older children. The impact of exposure to the toxin is assessed as low to moderate depending on the age of the child. Large‐scale control measures were implemented to rapidly withdraw contaminated infant formula products in the EU and recalls are ongoing. As a result, the current likelihood of exposure is considered low. However additional cases may still occur as recalled products may remain in households.

Suggested citation: European Centre for Disease Prevention and Control, European Food Safety Authority, 2026. Multi-country foodborne event caused by cereulide in infant formula products – 19 February 2026.

Catalogue number: TQ-01-26-008-EN-N; ISBN: 978-92-9498-863-8; DPO: 10.2900/7243107

Also published in the EFSA Journal: Scientific report approved by EFSA on 19 February 2026; doi:10.2903/j.efsa.2026.9984; Key words: cereulide, *Bacillus cereus*, multi-country recall, infant formula products, food incident. Requestor: European Commission; Question number: EFSA-Q-2026-00085; correspondence: Ask a question, ISSN: 1831-4732.

## Event background

On the 19 December 2025, Denmark reported receiving signals of gastrointestinal disease in infants after the recall of infant formula initiated in December (event ID 2025-FWD-00107 EpiPulse, the European Surveillance Portal for Infectious Diseases). Countries were asked to share information about their investigations following the recall to identify a possible link between the recalled infant formula batches and cases of gastrointestinal disease in children, and to assess the public health impact of the event. The recall was undertaken due to a contamination with cereulide, a toxin produced by *Bacillus cereus*.

*B. cereus* can cause two forms of toxin-mediated illness: an emetic form (nausea and vomiting) and a diarrhoeal form. The emetic form is caused by cereulide, a highly thermostable toxin produced in food before consumption and implicated in the present event. Cereulide causes sudden onset of nausea and vomiting shortly after ingestion. The incubation period ranges from 30 minutes to six to eight hours.

Diagnosis of the emetic type relies on identifying cereulide rather than detecting the bacterium itself. Food poisoning caused by *B. cereus* toxin, regardless of type, is not a notifiable disease in most countries in the EU/EEA. However, reporting of foodborne outbreaks to EFSA with aggregated information is possible (Annex 2). Cereulide toxin analysis in faecal samples is not a method that is available for routine diagnostic purposes in clinical microbiological laboratories. Instead, in foodborne outbreaks caused by cereulide, outbreak cases are often confirmed by an epidemiological link, i.e. linking symptoms of cereulide intoxication to consumption of a specific food item that tests positive for the toxin.

The diagnostic challenges and limited surveillance affect the ability of countries to confirm human cases in the current event. In addition, intoxication with cereulide produces symptoms that closely most often resemble those of viral gastrointestinal infections, which are common in Europe at this time of year. However, viral gastroenteritis often spreads easily within households and leads to secondary cases, whereas cereulide intoxication does not.

## Epidemiological and microbiological investigations of human cases

As of 13 February 2026, investigations were still ongoing in European countries to assess the possible link between consumption of infant formula products from recalled batches, and cases of gastrointestinal disease in infants. Cases were reported using national criteria for case classification and association of illness to implicated products. The European case classification is still under development.

Information to ECDC from the seven countries that have reported observing infants with symptoms of cereulide intoxication following consumption of infant formula products is summarised below.

The majority of infants have presented with mild symptoms, but hospitalisations due to dehydration have also been reported. The diagnostic challenges and limited surveillance affect the ability of countries to confirm human cases in this event. The latest disease onset that has been reported to ECDC was on 6 February 2026 with public health investigations still ongoing in countries.

**Austria** reported four infants who developed gastrointestinal symptoms, particularly vomiting, shortly after consuming infant formula. For one infant, the cereulide was detected in the infant formula consumed, two infants, consumed products which were part of a recalled batch, and one child had consumed formula from a non-recalled batch. The children, aged between 0 and 11 months of age, fell ill between 26 January and 6 February 2026 and one child was hospitalised. All children have now recovered.

**Belgium** reported eight infants who tested positive based on clinical samples. All eight had consumed the recalled infant formula and experienced favourable clinical outcomes. Cereulide was detected in the formula products consumed by five of the infants.

**Denmark** reported having no diagnostics for human samples and thereby no surveillance of confirmed cases. Suspected cases are not captured in the surveillance system. The food safety authority in Denmark has been contacted by parents of approximately 32 infants who developed gastrointestinal symptoms (self-reported) after the recall was initiated in December 2025. Some of the infants had consumed the batches of infant formula that was part of the recall, but some had consumed other batches.

**France** reported 11 infants who were hospitalised and under investigation. All infants have recovered and have returned home. Five of them consumed recalled infant formula, and it was not possible to confirm consumption for the remaining six children. Five of the 11 children who were hospitalised received a differential diagnosis for the gastro-enteritis symptoms. Two unexplained infant deaths were reported in France and are currently under routine medico-legal investigation. Although both infants had consumed infant formula that was part of the recall, current investigations have not identified any link between the consumed formula and the illnesses or deaths.

**Luxembourg** reported three infants who were examined as part of this event. Two infants were hospitalised for dehydration and have fully recovered. Clinical samples were negative for all three infants, but the formula consumed by one infant was positive however the incubation time for this infant was 48 hours.

**Spain** reported 41 infants with gastrointestinal symptoms, all of whom had a history of consuming products which were part of the recall. Twelve of the 41 infants were hospitalised and have been discharged. Ten additional notifications of infants with compatible symptoms were also received, of whom nine had consumed unknown batches of infant formula, and one had consumed formula from batches not part of the recall. Of these ten infants, one was hospitalised and been discharged.

**The United Kingdom** reported 44 infants with gastrointestinal symptoms following the consumption of formula which was part of the implicated batches. The testing of recalled formula has confirmed the presence of cereulide.

## Microbiological and environmental investigations of food, and control measures implemented

This section summarises the food investigations and control measures implemented by the countries concerned by the detection of cereulide in infant formula products, as reported under the 13 relevant Rapid Alert System for Food and Feed (RASFF) notifications as of 4 February 2026. Annex 1 provides a detailed summary of the traceability data shared by the involved countries per RASFF notification.

A multi-country recall of several infant formula products (various brands and batches) from different food companies is ongoing in Europe following the detection of cereulide. The recall is global and was initiated in December 2025, continued into January 2026, and further expanded in February 2026 following the publication of an EFSA Rapid Risk Assessment on 2 February 2026. Based on this, EFSA estimated concerning levels of cereulide in infant formula products. This EFSA Rapid Risk Assessment was intended to help EU risk managers determine when products should be withdrawn from the market as a public health measure[1].

The cereulide contamination of the infant nutrition products was first identified by the Swiss Company A in its manufacturing plant in the Netherlands (the Dutch Manufacturer A) in late November 2025. The contamination of the infant formula products with cereulide was traced back to a contaminated ingredient, namely the arachidonic acid (ARA) oil, manufactured by the Chinese Producer A (*fup56,* 2025.9962) and used in the concerned infant formula products (*fup28, fup32,* 2025.9962). The Dutch Manufacturer A had supplied the contaminated ingredient via the Swiss Producer B. The Swiss Producer B had received the contaminated ARA oil from the Chinese Producer A and had used it for the preparation of the oil mixes which were further delivered to the different manufacturers of the Swiss Company A, including the Dutch Manufacturer A. The Swiss Producer B carried out a retrospective investigation to trace the contamination back and analysed all ARA oil deliveries received from the Chinese Producer A between April 2023 and October 2025 (*fup4*, 2026.0177). The contamination was dated back to October 2024 and persisted throughout 2025, with the highest concentration observed in deliveries in July 2025 (*fup4*, *fup12*, 2026.0177).

The detection of cereulide in the infant formula products led to an initial precautionary recall that was executed by the Swiss Company A on 10 December 2025. The Swiss Company A informed the national food safety authorities and the European Commission, and issued a public recall, contacted its manufacturing plants that had received the concerned ARA oil (*fup29, fup32, fup82,* 2025.9962) and corresponding oil mixes, and blocked the supplies from the Chinese Producer A (*fup96* 2025.9962). In January 2026, the European Commission requested that the International Food Safety Authorities Network (INFOSAN) Secretariat contact the Chinese authorities to provide information about the contaminated batches of ARA oil from the Chinese Producer A, as well as their distribution, the complete root cause analysis, and remedial action taken. As of 13 February 2026, this information had not been provided by the Chinese authorities (*fup10,* 2026.0177; *fup14*, 2026.0177).

This first identification of the contamination of the ARA oil (and corresponding oil mixes) with cereulide triggered further food investigations in the food sector. At the time of publication, eight food companies (and their related manufacturing plants) had received the ARA oil and initiated a recall from the market of their infant nutrition products containing batches of ARA oil potentially contaminated with cereulide. Based on the traceability information available from RASFF, the majority of these food companies had received contaminated batches of ARA oil (and corresponding oil mixes) via different suppliers with trading relations with the Chinese Producer A.

[1] EFSA (European Food Safety Authority), Eskes, C., Cortiñas-Abrahantes, J., Bottex, B., Dorne, J.L.C.M., Dujardin, B., de Souza, R.F., Horvath, Z., Kouloura, E., Bordajandi, L.R., Rizzi, V., Steinkellner, H., Gilsenan, M. Rapid risk assessment on acute reference dose (ARfD) of cereulide in infants and information on acute consumption of infant formulae. EFSA Journal, 24(1), e9941, https://doi.org/10.2903/j.efsa.2026.9941

The food safety authority in Belgium confirmed that four samples of infant formula products consumed by infants tested positive for the cereulide. The products which are expiring in 2027 belonged to the Swiss Company A (Product M Batch P; Product N Batch Q) and to the French Company E (Product O Batch R and Batch S).

The food safety authority in Luxembourg confirmed that an infant formula product consumed by an infant tested positive for the cereulide. The product which expires in 2027 belonged to Swiss Company A and it was Product M Batch P (*fup36*, 2025.9962). This product tested also positive in Belgium.

The food safety authority in Austria confirmed that an infant formula product consumed by an infant tested positive for the cereulide. The product expiring in 2027 belonged to the French Company E (Product P Batch T) (*fup17*, 2026.0663). In addition, the authority clarified that the two infant formula products consumed by the cases and belonging to batches know to contain cereulide were Product Q Batch U and Batch V, and Product R Batch W and Batch X. The products expiring in 2026 and 2027 belonged to the French Company E.

The national food safety authorities in the European countries where the infant nutritional products were distributed, reported the outcome of their traceability investigations and the implementation of several control measures implemented including the withdrawal and the recall of the concerned products in RASFF, and public warnings issued to inform consumers.

## ECDC and EFSA risk assessment for the EU/EEA

As of 13 February 2026, six EU countries (Austria, Belgium, Denmark, France, Luxembourg, and Spain), as well as the United Kingdom, reported investigating the source of gastrointestinal symptoms in infants and the consumption of infant formula products. A global recall began in December 2025 as a preventive public health measure, continued in January 2026, and were expanded in February 2026 to include additional products that were unlikely to meet the calculated cereulide concentration thresholds (in reconstituted (liquid) infant formula) based on the EFSA Rapid Risk Assessment published on 2 February 2026. As of that date, the recall within the EU was harmonised under a science-based risk management approach. The root cause analysis of the infant formula products contaminated with cereulide carried out by the Swiss Company A in November 2025, identified the ARA oil ingredient from the Chinese Producer A, and triggered food investigations in December 2025 by several other companies that used the same ARA oil.

The risk of exposure to contaminated products in the EU has been significantly reduced by the large-scale and ongoing recall, the interruption of trading the contaminated ARA oil ingredient, and public warnings implemented by the concerned companies and the national food safety authorities where products were distributed.

In Belgium, as of 10 February 2026, the possible vehicle of illness was identified when both the consumed infant formula products and the clinical samples tested positive for cereulide. The products belonged to the Swiss Company A and the French Company E. Among those, the batch of infant formula products from the Swiss Company A reported in Belgium was also consumed by an infant in Luxembourg whose clinical sample was negative and where the incubation time was 48 hours. In Austria, clinical symptoms from patients that had consumed infant formula products, testing positive for cereulide (or belonging to batches testing positive) from the French Company E, supports the hypothesis of these products as possible vehicle of gastrointestinal illness. Investigations from national public health authorities and food safety authorities are ongoing to verify the hypothesis of recalled batches or other batches of infant formula products as vehicle of gastrointestinal illness.

Cereulide intoxication normally presents with relatively mild symptoms, but neonates and young infants less than six months of age may be more likely to develop more severe symptoms and are also more sensitive to dehydration, electrolyte abnormalities, etc. The impact of exposure to the toxin is therefore assessed as low to moderate, depending on the age of the child.

As a result of the recall, which is still ongoing, the current likelihood of exposure is considered low. However, if products subjected to the recall are retained by consumers rather than returned to the point of sale, they can still constitute a risk of exposure and might result in additional cases. The national public health authorities and food safety authorities are following up with their investigations. ECDC and EFSA continue to monitor the event, and a European case definition is under development.

## Recommendations

ECDC and EFSA recommend the following actions for public health authorities, food safety authorities, and consumers in response to ongoing investigations:

**Public health authorities** are encouraged to share information about cases and their investigations with ECDC and other European countries via the platform EpiPulse, using the EU case definition when it has been finalised and shared in EpiPulse, and to work closely with food safety authorities to investigate infants with symptoms of cereulide intoxication and to assess whether recalled batches or additional batches of infant formula products may have been the vehicle.

**Food safety authorities** are advised to follow the recommendation of the European Commission regarding the contamination thresholds, as calculated based on the EFSA Rapid Risk Assessments (https://efsa.onlinelibrary.wiley.com/doi/10.2903/j.efsa.2026.9941); and to share the outcome of their national food investigations in RASFF.

**Consumers** are advised to follow the instructions and guidance issued by national food safety authorities. Recalled products should not be given to infants or young children and returned to the point of sale. It is important to be attentive to symptoms of vomiting and diarrhoea in infants and young children, regardless of their underlying cause, and the recommendation is to seek professional medical advice if infants or young children develop persistent or severe gastro-intestinal symptoms.

## Source and date of request

ECDC sent a request to EFSA on 28 January 2026 to produce a Joint Rapid Outbreak Assessment (ROA). EFSA accepted the request on 28 January 2026.

## Consulted experts and national contact points

**ECDC experts (in alphabetical order):** Céline Gossner, Cecilia Jernberg, Nathalie Jourdan-da Silva, Elena Portell Buj and Linda Trönnberg

**EFSA staff (in alphabetical order):** Laura Cabral, Valentina Rizzi, Eleonora Sarno, Frank Verdonck.

**Public health experts consulted for data and facts validation:**

**Austria:** Sabine Maritschnik (Austrian Agency for Health and Food Safety) and Dirk Werber (Austrian Agency for Health and Food Safety).

**Belgium**: Dieter Van Cauteren (Sciensano) and Géraldine De Muylder (Sciensano).

**Denmark**: Luise Müller (head of the outbreak unit in the Section of Food- and Waterborne and Zoonotic Infections at the Department of Infectious Disease Epidemiology and Prevention) and Aoife Ronayne (head of the bacterial special diagnostics laboratory at the Department of Bacteria, Parasites and Fungi).

**France**: Gabrielle Jones (Santé Publique France), Geneviève Buser (Santé Publique France) and Henriette De Valk (Santé Publique France).

**Luxembourg**: Anne Vergison (Health Inspection), Joël Mossong (Health Inspection), Rafaëla Schober (Health Inspection), Corinna Ernst (Health Inspection), Théo Duhamel (Health Inspection), Isabelle Mootz (Health Inspection), Emilie Culetto (Health Inspection), Telma Velez (Health Inspection), Isabelle Kerrec (Health Inspection), and Isabel De La Fuente (Centre Hospitalier du Luxembourg).

**Spain**: Carmen Varela Martinez (National Centre of Epidemiology, ISCIII), Silvia Herrera León (National Centre of Microbiology, ISCIII) and Esteban Aznar Cano (European Programme for Intervention Epidemiology Training-EPIET, Alert and Emergency Coordination Centre, Ministry of Health).

**The UK**: Vanessa Wong, Trish Mannes, Gauri Godbole, Nicola Love, Ella Rodwell, Thomas Thackray (UK Health Security Agency), Andrew Nelson, Chris Williams (Public Health Wales), Genna Leckenby (Public Health Scotland) and Patrick McAleavey (Public Health Agency Northern Ireland)

**RASFF contact points consulted:** Austria, Belgium, Bulgaria, Croatia, Cyprus, Czech Republic, Denmark, Estonia, Finland, France, Germany, Greece, Hungary, Iceland, Ireland, Italy, Latvia, Liechtenstein, Lithuania, Luxembourg, Malta, Netherlands, Norway, Poland, Portugal, Romania, Slovakia, Slovenia, Spain, Sweden, Switzerland.

**National experts consulted by the RASFF contact points:**

**Austria**: Christoph Czerwenka, Bernhard Kuhn, Hans Steinwider (AGES - Austrian Agency for Health and Food Safety).

**Croatia**: Nikolina Grizelj, Alen Petričević, Vlatka Erman Oštrić, Romana Franić (State Inspectorate, DIRH - NCP); Brigita Hengl; Jasenka Petrić; Danijela Stražanac (Croatian Agency for Agriculture and Food, HAPIH).

**Denmark**: Anne Ribert Larsen, Tanja Østergaard Pedersen, Danish Veterinary, Food, Agriculture and Fisheries Agency.

**Finland**: Mika Varjonen and Paula Hietanen (Finnish Food Authority).

**France**: Caroline Kojfer-Lomont and Lorraine Puzin (Emergency Unit, Ministry of Agriculture, Agri-Food and Food Sovereignty).

**Ireland**: Karen McCullagh, Michelle Minihan, Orla Moore, and Shaun Smith (Food Safety Authority of Ireland).

**Italy:** Raffaello Lena, Valentina Cambiotti, Eleonora Chelli (Italian Ministry of Health).

**Luxembourg**: Claude Scholtes and Greet Symons (ALVA, Luxembourg Veterinary and Food Administration).

**Switzerland**: Manel Nobel and Andrea Blank, Federal Food Safety and Veterinary Office.

**The Netherlands**: Huib Schillings and Coen Van der Weijden (Netherlands Food and Consumer Product Safety Authority, NVWA).

## Disclaimer

This rapid outbreak assessment was written jointly by the European Centre for Disease Prevention and Control (ECDC) and the European Food Safety Authority (EFSA).

ECDC issued this outbreak assessment document in accordance with Article 20 of Regulation (EU) 2022/2371 on serious cross-border threats to health, Articles 7(1) and 8a of Regulation (EC) No 851/2004 establishing a European Centre for Disease Prevention and Control. EFSA's contribution is based on a mandate from the European Commission requesting EFSA to provide scientific assistance from EFSA in the investigation of multinational food-borne outbreaks (Ares (2013) 2576387, Mandate M-2013-0119, 4 July 2013) in accordance with Article 31 of Regulation (EC) No 178/2002 of the European Parliament and of the Council of 28 January 2002, laying down the general principles and requirements of food law, establishing the European Food Safety Authority and laying down procedures in matters of food safety.

The specific purpose of an ECDC-EFSA rapid outbreak assessment is to present an analysis of a cross-border food-borne threat to health and to provide science-based recommendations and options for response. The responsibility for the choice of which options to pursue and which actions to take at national level, following ECDC and EFSA's recommendations, lies with EU/EEA countries.

All data published in this rapid outbreak assessment are data collected from EU and/or EEA countries concerned by the outbreak until the date this assessment was produced. Maps and figures published do not represent statements from ECDC or EFSA on the legal or border status of the countries and territories shown but constitute the information on which this rapid outbreak assessment is based.

## Annex 1. Description of the traceability, food investigations, and control measures by country

This section summarises the food investigations and control measures implemented by the countries concerned by this food incident as reported under the relevant RASFF notifications. As of 4 February 2026, there were 13 RASFF notifications issued by eight countries: 2025.9962 (contained 106 European Commission (EC) validated follows up (*fup*) and 172 Member States (MS) validated *fup*; 2026.0027 (contained 39 EC validated *fup*); 2026.0173 (contained 9 EC validated *fup*); 2026.0179 (19 EC validated *fup*); 2026.0196 (contained 12 EC validated *fup*); 2026.0542 (contained 5 EC validated *fup*); 2026.0177 (contained 13 EC validated *fup*); 2026.0407 (contained 6 EC validated *fup*); 2026.0347 (contained 9 EC validated *fup*); 2026.0509 (contained 15 EC validated *fup*); 2026.0598 (contained 23 EC validated *fup*); 2026.0633 (contained 21 EC validated *fup*); 2026.0647 (contained 15 EC validated *fup*).

**Swiss Food Company A**

**2025.9962 Dutch Manufacturer A**

On 10 December 2025, the food safety authority in Italy issued the first RASFF notification (2025.9962) linked to the cereulide contamination incident to report the measures they implemented consisting in the recall from the market of some infant formula products (Product A Batch A and Product B Batch B) originating from the Dutch Manufacturer A. This measure followed the detection of the cereulide during an own check control (*fup8*, 2025.9962) at the Dutch Manufacturer A that belongs to the Swiss Company A.

On 6 January 2026, the European Commission informed in RASFF (*fup28*, 2025.9962) to have been in contact with the Swiss Company A and the food safety authority in the Netherlands. The Swiss Company A had directly informed the European Commission and the food safety authority in the Netherlands about the cereulide contamination incident. Specifically, in late November 2025, the Dutch Manufacturer A received first results indicating the cereulide contamination of some products not yet distributed to the market and informed the food safety authority about the root cause analysis carried out. The cereulide contamination was traced back to the arachidonic acid (ARA) oil manufactured by the Chinese Producer A (*fup56,* 2025.9962) and supplied by the Belgian Supplier A. The ARA oil (and corresponding oil mixes containing it) was the contaminated ingredient (*fup56,* 2025.9962) used in the concerned infant formula products (*fup28, fup32,* 2025.9962). The cereulide detection led to an initial precautionary recall that was executed by the Swiss Company A on 10 December 2025 (25 batches distributed across 15 different European markets). Specifically, the Swiss Company A informed the authorities, issued the public recall, contacted its manufacturing plants that had received the concerned ARA oil (*fup29, fup32, fup82,* 2025.9962) and corresponding oil mixes, and interrupted the import from the Chinese Producer A (*fup96* 2025.9962).

On 9 January 2026, the European Commission informed in RASFF (*fup48,* 2025.9962) about the outcome of the investigation retrospectively done by the Swiss Company A to determine the extent of the potential contamination in the finished products. Overall, 65 batches of ARA oil (and corresponding oil mixes) received between April 2023 - December 2025 were analysed (ISO 18465:2017). The results indicated that contamination was first detected in ARA oil deliveries received at the end of 2024.

The Swiss Company A therefore recalled all the batches of infant formula products with a level of cereulide above or equal to 0.2 ng/g (ISO 18465:2017) (*fup48,* 2025.9962).

The recall of the infant formula products was further extended worldwide (*fup34, fup37,* 2025.9962). As of 4 February 2026, overall, 13 RASFF notifications had been issued to report the traceability information of the batches concerned and the consequent control measures implemented by the countries (in the EU and outside) concerned by the global distribution, including the engagement of the national food safety authorities with the national manufacturing plants of the Swiss Company A.

On 2 February 2026, the food safety authority in the Netherlands informed in RASFF that the Dutch Manufacturer A of the Swiss Company A had decided to extend the recall to batches that were considered negative in December 2025 (according to ISO 18465:2017 in milk powder without reconstitution) that following calculations of ARA oil/oil mix were considered probably not to meet the EU action limits[2] (*fup98*, *fup100*, 2025.9962).

[2] EFSA (European Food Safety Authority), Eskes, C., Cortiñas-Abrahantes, J., Bottex, B., Dorne, J.L.C.M., Dujardin, B., de Souza, R.F., Horvath, Z., Kouloura, E., Bordajandi, L.R., Rizzi, V., Steinkellner, H., Gilsenan, M. Rapid risk assessment on acute reference dose (ARfD) of cereulide in infants and information on acute consumption of infant formulae. EFSA Journal, 24(1), e9941, https://doi.org/10.2903/j.efsa.2026.9941 dose (ARfD) of cereulide in infants and information on acute consumption of infant formulae. EFSA Journal, 24(1), e9941, https://doi.org/10.2903/j.efsa.2026.9941

**2026.0027 German Manufacturer B**

On 5 January 2026, the food safety authority in Austria notified in RASFF (2026.0027) the detection of cereulide in some infant formula products (Product C Batch C and Product D Batch D) from the German Manufacturer B of the Swiss Company A (*fup31*, 2026.0027). The products were sampled in official control at retail level on 16 December 2025.

On 9 January 2026, the food safety authority in Germany informed in RASFF that on 5 January 2025 the German Manufacturer B had initiated the recall of the products (different products, brands, and batches) that had been distributed in the EU and outside (*fup4,* 2026.0027). The countries that had received the batches concerned by the recall reported in RASFF the outcome of the investigations and the relative measures taken.

**2026.0173 Spanish Manufacturer C**

On 8 January 2026, the food safety authority in Spain reported in RASFF (2026.0173) that the Spanish Manufacturer C of the Swiss Company A had implemented the recall from the market of their infant formula products (different products, brands, and batches) concerned by the contaminated batches of ARA oil (and corresponding oil mixes), and distributed in the EU and outside. The countries that had received the batches concerned by the recall reported in RASFF the outcome of the investigations and the relative measures taken.

**2026.0179 Swiss Manufacturer D**

On 8 January 2026, the food safety authority in Switzerland reported in RASFF (2026.0179) that the Swiss Manufacturer D of the Swiss Company A had implemented the recall from the market of their infant formula products (different products, brands, and batches) concerned by the contaminated batches of ARA oil (and corresponding oil mixes), and distributed in the EU and outside. The countries that had received the batches concerned by the recall reported in RASFF the outcome of the investigations and the relative measures taken.

**2026.0196 French Manufacturer E**

On 12 January 2026, the food safety authority in France reported in RASFF (2026.0196) that the French Manufacturer E of the Swiss Company A had implemented the recall from the market of their infant formula products (different products, brands, and batches) concerned by the contaminated batches of ARA oil (and corresponding oil mixes), and distributed in the EU and outside. The countries that had received the batches concerned by the recall reported in RASFF the outcome of the investigations and the relative measures taken. The food safety authority in France has further requested to the concerned company to give proof of its compliance with the cereulide concentration thresholds (in reconstituted (liquid) infant formula) calculated based on the EFSA Rapid Risk Assessment and further published on 2 February 2026.

**2026.0542 Dutch Manufacturer A**

On 22 January 2026, the food safety authorities in Croatia reported in RASFF (2026.0542) the implementation of the recall from the market of some infant formula products (Product E Batch E) produced by the Dutch Manufacturer A of the Swiss Company A. Product E Batch E had been sampled on 16 December 2025 at retail level during an official control and tested cereulide positive. The concerned products had been distributed in the EU (*fup1*, *fup2*, 2026.0542).

On 29 January 2026, the food safety authority in the Netherlands informed that the Dutch Manufacturer A was notified by the Croatian authorities about the Product E Batch E, and a recall was initiated (*fup2*, 2026.0542).

On 4 February 2026, the food safety authorities in Luxembourg reported in RASFF (2026.0542) the implementation of the recall from the market of some batches of an infant formula product (Product L) produced by the Dutch Manufacturer A of the Swiss Company A (*fup100*, 2025.9962). The food safety authority informed to have carried out checks and that the results for this product were non-compliant. The Swiss Company A then decided to recall the product via its plant (*fup4*, 2026.0542).

**2026.0177 Swiss Producer B**

On 8 January 2026 the food safety authority in Switzerland reported in RASFF (2026.0177) the detection of cereulide (ISO 18465:2017) in some batches (Batch F and Batch G) of oil mixes (containing ARA oil). The products were sampled on 30 December 2025 in own check at the Swiss Producer B.

The Swiss Producer B had received the contaminated ARA oil from the Chinese Producer A and had used it for the preparation of the oil mixes further delivered to the different manufacturers of Swiss Company A. Additionally, the food safety authority in Switzerland clarified that the other final products produced at the Swiss Producer B were not concerned by the cereulide contamination.

The Swiss Producer B carried out a retrospective investigation to trace the contamination back and analysed all ARA oil deliveries received from the Chinese Producer A between April 2023 and October 2025 (*fup4*, 2026.0177). The contamination of the raw material was dated back to end of 2024 (October 2024) and persisted throughout 2025 with the highest concentration observed in deliveries in July 2025 (*fup4*, *fup12*, 2026.0177).

As of 5 January 2026, the Swiss Producer B implemented a mandatory cereulide testing for all ARA oil (and corresponding oil mixes) deliveries (including from other suppliers of fermented oils). Only raw material and finished oil mixes testing <0.5 ng/g were released (*fup4*, 2026.0177).

The ARA oil (and corresponding oil mixes) had been delivered worldwide to the manufacturers of Swiss Company A, e.g. in South Africa, Argentina, Brazil, Germany, Spain, France, India, the Netherlands, Philippine, Switzerland, and China (*fup12*, 2026.0177).

In January 2026, the European Commission informed to have requested the INFOSAN Secretariat to contact the Chinese authorities to provide information about the contaminated batches of ARA oil from the Chinese Producer A and their distribution, about the complete root cause analysis, and remedial action taken. This information was not provided by the Chinese authorities (*fup10,* 2026.0177; *fup14*, 2026.0177).

**Italian Food Company B**

**2026.0407**

On 9 January 2026, the food safety authority in Italy issued a RASFF notification (2026.0407) to inform that the Italian Company B had initiated the voluntary recall from the market of three batches of infant follow-on formulae (Product F Batch H, Batch I, and Batch N). Two batches (Batch H, Batch I) of infant products contained a cereulide contaminated batch (Batch J) of ARA oil received from the Dutch Producer C (*fup4*, *fup6*, 2026.0407). One batch (Batch N) of infant products contained a batch (Batch O) of ARA oil that tested negative according to the analysis done by the Dutch Producer C.

Product F had been distributed within Italy and to Vatican City (*fup2 fup3*, 2026.0407).

**Swiss Food Company C**

**2026.0347**

On 15 January 2026, the food safety authority in Switzerland informed in RASFF (2026.0347) that the Swiss Company C had implemented the precautionary recall from the market of the infant formula products produced for its three customers using batches of ARA oil (Product G) contaminated with cereulide and originating from the Chinese Producer A. The three customers further delivered the infant formula products to different countries.

The Swiss Company C supplied the contaminated batches of ARA oil (Product G) from the Chinese Producer A (*fup2*, 2026.0347) via the German Supplier B that distributed to its four recipients. Upon identification of the contamination incident, the German Supplier B blocked the existing stocks and informed the recipients including the Swiss Company C (*fup2*, 2026.0347), the Austrian Company F, the French Company G, and the Dutch Producer C (*fup2*, 2026.0347).

The Dutch Producer C used the batches of contaminated ARA oil (Product G) supplied by the German Supplier B to produce oil mix (Product H) that was further distributed in EU and outside the EU (*fup4*, 2026.0347) including to the Italian Company B that initiated the precautionary recall of its infant formula products described earlier in 2026.0407 (*fup5*, 2026.0347).

The countries concerned by the distribution of the oil mix (Product H) reported in RASFF the outcome of their traceability investigations and measures taken.

**French Food Company D**

**2026.0509**

On 21 January 2026, the food safety authority in France informed in RASFF (2026.0509) that the French Company D had implemented the recall of different batches of infant formula products produced and distributed worldwide. The French Company D received via the Dutch Producer C batches of oil mix (Product H) containing contaminated ARA oil. The countries concerned by the distribution reported in RASFF the outcome of their traceability investigations and measures taken. The food safety authority in France has further requested to the concerned company to give proof of its compliance with the cereulide concentration thresholds (in reconstituted (liquid) infant formula) calculated based on the EFSA Rapid Risk Assessment and further published on 2 February 2026.

**French Food Company E**

**2026.0598 Irish Manufacturer F**

On 23 January 2026, the food safety authority in Ireland reported in RASFF (2026.0598) the implementation of the recall of 16 batches of various infant formula products from the Irish Manufacturer F of the French Company E. This measure was taken following the detection of cereulide in the final products during an own check control (2026.0598). Some batches of infant formula were produced at an Irish manufacturing site whilst others were incorporated into finished products in manufacturing facilities in other Member States where the contaminated base powder from Ireland was used as an ingredient (*fup8*, 2026.0598). The infant formula products had been distributed in EU and outside.

The countries concerned by the distribution reported in RASFF the outcome of their traceability investigations and the measures taken.

The food safety authority in France has further requested to the French Company E to give proof of its compliance with the cereulide concentration thresholds (in reconstituted (liquid) infant formula) calculated based on the EFSA Rapid Risk Assessment and further published on 2 February 2026.

**2026.0663 German Manufacturer G**

On 26 January 2026, the food safety authority in Germany reported in RASFF (2026.0663) that the German Manufacturer G of the French Company E implemented the withdrawal of three products that had been produced using the contaminated base powder from Ireland. On 30 January 2026, the withdrawal was extended to a public recall (*fup13*, 2026.0663). Specifically, Product I Batch K was produced for the German Manufacturer G at the plant in Ireland (Plant A) and Product J Batch L was produced at the plant in Polan (Plant B). Only Product K Batch M was manufactured at the German Manufacturer G. The three concerned batches were distributed in EU and outside.

On 6 February 2026 the food safety authority in Germany informed in RASFF that the German Manufacturer G had further extended the precautionary recall to additional products and batches (*fup24*, 2026.0663).

The countries concerned by the distribution reported in RASFF the outcome of their traceability investigations and the measures taken.

**The French Company G and French Food Company H**

**2026.0647**

On 27 January 2026, the food safety authority in France informed in RASFF (2026.0647) that the French Company H and the French Company G had implemented the recall of different batches of infant formula products produced and distributed in EU and outside the EU. The French Company G had received via the German Supplier B batches of oil mix (Product G) containing contaminated ARA oil and then produced infant formula products for the French Food Company H.

The countries concerned by the distribution reported in RASFF the outcome of their traceability investigations and measures taken.

The food safety authority in France has further requested the concerned company to give proof of its compliance with the cereulide concentration thresholds (in reconstituted (liquid) infant formula) calculated based on the EFSA Rapid Risk Assessment and further published on 2 February 2026.

## Annex 2. Food-borne outbreaks caused by *Bacillus cereus*

Country-specific data on food-borne outbreaks are reported to EFSA by countries in accordance with the Zoonoses Directive 2003/99/EC. Overall, one strong-evidence foodborne outbreak caused by *Bacillus cereus* (but with quantification of cereulide) was reported by Romania in 2015 with the reported food vehicle 'Milk, cows' - pasteurised milk' that represents the closest food category to the infant formula food products in this food incident. The outbreak is reported to be associated to setting 'school or kindergarten'. There were 23 human cases, 21 hospitalisations, and no deaths reported.
